# Dietary Intake among Lebanese Adults: Findings from the Updated LEBANese natiONal Food Consumption Survey (LEBANON-FCS)

**DOI:** 10.3390/nu16111784

**Published:** 2024-06-06

**Authors:** Maha Hoteit, Maroun Khattar, Dana Malli, Esraa Antar, Zahraa Al Hassani, Maher Abdallah, Dalia Hachem, Elham Al Manasfi, Abdulrahman Chahine, Nikolaos Tzenios, the Adults-LEBANON-FCS Group

**Affiliations:** 1 Food Sciences Unit, National Council for Scientific Research-Lebanon (CNRS-L), Beirut P.O. Box 11-8281, Lebanon; 2 Faculty of Public Health, Section 1, Lebanese University, Beirut P.O. Box 6573, Lebanondana.malli@st.ul.edu.lb (D.M.); e.antar@st.ul.edu.lb (E.A.); zahraa.al-hassani@st.ul.edu.lb (Z.A.H.);; 3 Arab Group for Scientific Research, Beirut 1103, Lebanon; 4 Faculty of Public Health, Charisma University, London EC1V 7QE, UK

**Keywords:** adult, food consumption, food groups, macronutrients, micronutrients, healthy diets

## Abstract

Background: The rates of obesity, undernutrition, and other non-communicable diseases are on the rise among Lebanese adults. Therefore, it is crucial to evaluate the food consumption habits of this population to understand diet quality, analyze consumption trends, and compare them to healthy diets known to reduce risks of non-communicable diseases. Aim: To evaluate the food consumption patterns, energy intake, as well as macro- and micro-nutrient intake among a nationally representative sample of Lebanese adults aged 18−64 years old. Methods: A cross-sectional study was carried out from May to September 2022 involving 444 participants from all eight Lebanese governorates. Sociodemographic and medical information was gathered through a questionnaire, food consumption was evaluated using a validated FFQ and 24 h recall, and anthropometric measurements were recorded. Results: There was a notable lack of adherence to three healthy diets (Mediterranean, EAT-Lancet, USDA) among Lebanese adults. Their dietary pattern is characterized by high energy, added sugars, sodium, and saturated fat intake while being low in healthy fats, vitamin A, D, and E. Adult women are falling short of meeting their daily calcium, vitamin D, iron, and vitamin B12 requirements, putting them at increased risk of anemia, osteoporosis, and other health issues. Grains and cereals were the most consumed food groups, and most participants were found to be overweight or obese. Conclusions: In conclusion, the results highlight the need for public health policies and interventions aimed at encouraging Lebanese adults to make healthier food choices and transition towards diets like the Mediterranean, EAT-Lancet, or USDA diet. These diets have been shown to promote overall health and wellbeing.

## 1. Introduction

In an era marked by rapid globalization, urbanization, demographic and epidemiological transitions, and economic volatility, the dynamics of nutrition transition and food insecurity have become critical focal points for policymakers, researchers, and public health practitioners worldwide. The intricate interplay between nutrition transition—the shift in dietary patterns towards higher consumption of high-caloric food and a decrease in consumption of healthy foods [1]—and food insecurity—the inadequate access to sufficient, safe, and nutritious food—is emblematic of multifaceted challenges facing contemporary societies. This transition is often accompanied by a rise in sedentary lifestyles, reduced physical activity, and changing patterns of food preparation and consumption, heralding a new era of dietary diversity but also posing significant challenges to public health and nutrition. The current pattern of nutrition transition (consumption of high-caloric food and sedentary lifestyle) is characterized by the rising of nutrition-related non-communicable diseases (NR-NCDs) that seems to be accelerating in the lower- and middle-income transitional countries [2], which now carry more than 75% of the global non-communicable disease (NCD) burden [3]. This is the case in Lebanon, where Lebanese adults aged 18 years and older dietary patterns have shifted, and where the prevalences of obesity and undernourishment are increasing significantly. For instance, the prevalences of obesity (in 2016) and undernourishment (in 2020) increased and reached about 32% and 9.5%, respectively [4,5]. This is mainly due to the limited progress towards achieving the diet-related NCD targets, mainly obesity targets [6], although several diets were established and contributed to limit the development of NCDs, such as the Mediterranean, the EAT-Lancet and USDA diets, and dietary guidelines. This dietary shift is the result of changes the country has faced, and is still facing, since the past decade, such as increased urbanization, economic development, modernization in lifestyle, the COVID-19 pandemic, the massive blast that hit the Beirut port on 4 August 2020, and, most recently, the Ukraine–Russia war. The recent events have heightened the risk of food insecurity in the Lebanese population, affecting their nutritional behaviors and status [7,8,9]. This increased the risk of undernutrition, micronutrient deficiencies, and overweight/obesity in this population because of insufficient or imbalanced macro- and micro-nutrient intakes [10], which in turn can increase the risk of diet-related health conditions such as diabetes, hypertension, hyperlipidemia, and metabolic syndrome, among other conditions [11,12,13,14].

Given the significance of adopting healthy food choices to reduce the risk of non-communicable diseases, and with the rising rates of obesity, undernutrition, and other non-communicable diseases among Lebanese adults, it is essential to examine the dietary habits of this population. In addition, the current available data on adults’ food consumption in the country reflect the consumption of this population up to the year 2009, which, although important, is now outdated due to the changes that have occurred in the country since then and have affected food consumption. This highlights the importance of having new reliable data that reflect the recent trends of consumption in this population. Thus, this examination aims to understand the quality of Lebanese adults’ diets, analyze consumption trends, and compare them to proven healthy diets that can mitigate the risks of non-communicable diseases. By doing so, actionable steps can be taken to improve the dietary patterns of the population and eventually improve their overall health and wellbeing.

## 2. Methods

### 2.1. Study Design and Eligibility Criteria

This was a cross-sectional study that was conducted over a period of 5 months between May and September 2022 on a nationally representative sample of Lebanese adults. To be eligible, a participant had to be Lebanese and aged between 18 and 64 years old.

### 2.2. Sampling Method and Recruitment Process

**Figure 1:**
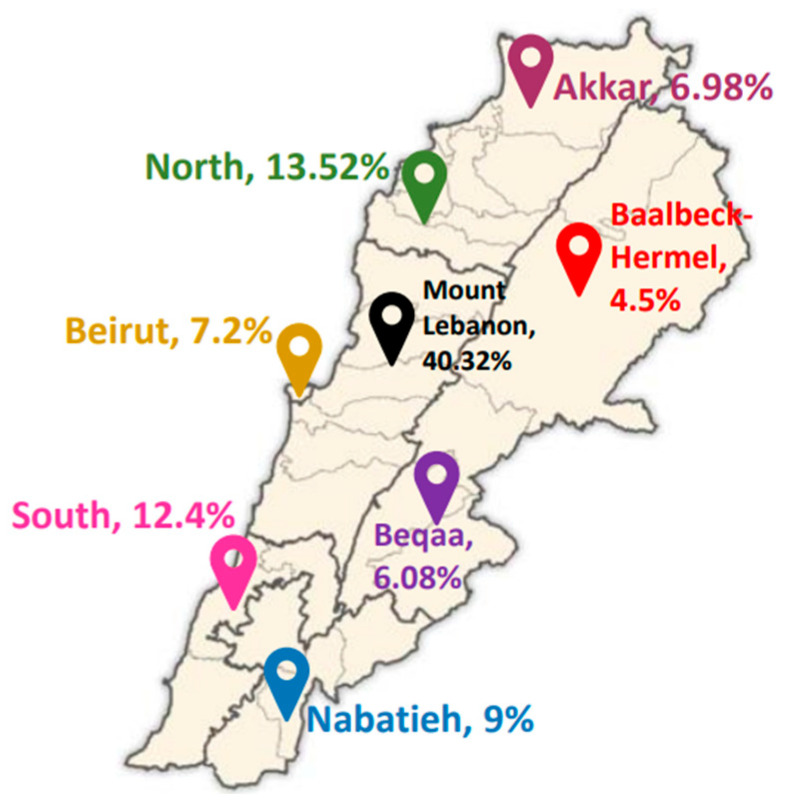
Participant distribution across the Lebanese governorates (Map template adapted from https://en.m.wikipedia.org/wiki/File:Lebanon_districts.png, accessed on 1 May 2024).

For the sample to be nationally representative, a minimum number of 400 participants was required. The sample size was calculated based on the population estimates from 2018 to 2019 using the following formula:

n = [p (1 − p)] × [(Z_∝/2_)^2^/(e)^2^]

where ‘n’ refers to the sample size; ‘Z_∝/2_’ refers to the standard error’s reliability coefficient at a 5% level of significance and is equal to 1.96; ‘p’ denotes the probability of adults (18–64 y) who were not capable of taking precautions regarding the diseases (50%); and ‘e’ represents the standard error’s tolerated level (5%), as stated by Hosmer and Lemeshow [15]. Overall, 449 participants (184 males and 265 females) from the 8 Lebanese governorates were included. The sampling technique was a combination of stratified cluster sampling, with the stratified groups being the two genders and the clusters being the Lebanese governorates. The process of recruiting participants involved various channels, including volunteers, charitable organizations, first-aid and medical centers, so a broad range of participants could be reached. Participants recruited in this stage were then encouraged to invite other individuals within their outreach to participate. This allowed us to reach participants that were difficult to reach physically due to budget constraints and timing. The individuals willing to participate were informed about the study nature and then assessed for eligibility. Eligible participants provided an electronic written consent indicating their willingness to participate in the study. One individual per Lebanese household was allowed to participate in the study so that a wide representation of households could be guaranteed. Overall, the study involved 444 participants, as 5 participants were excluded after checking for missing data, errors, and outliers. Participant distribution across the 8 governorates is shown in Figure 1.

### 2.3. Data Collection

#### 2.3.1. Phase 1: Administration of Sociodemographic Questionnaire

During the initial data collection phase, a pre-tested questionnaire was utilized in interviews with participants. This questionnaire covered demographic and socioeconomic details along with the medical background of the participants. For example, participants were queried about their age, gender, weight, height, place of residence, marital status, living space, household size, number of rooms in their residence, educational level, occupation, and any existing chronic illnesses. Questions pertaining to household size and number of rooms were included to compute the crowding index (CI), which serves as an indicator of a household’s socioeconomic standing [16].

#### 2.3.2. Phase 2: Food Frequency Questionnaire

Following the completion of the sociodemographic questionnaire, trained dietitians conducted 30-min interviews with participants to administer a 157-item semi-quantitative food frequency questionnaire (FFQ), which had been validated previously among the adult Lebanese population [17]. To assist the participants in remembering how much food they had consumed and in estimating portion sizes as precisely as possible, instructions and visual aids were given to them. The interviewers recorded how many servings and how often the stated portions (daily, weekly, or monthly) for each food item were consumed. Trained dietitians administered the FFQ. Furthermore, two 24 h recalls were conducted—one on a typical weekday and another on a weekend day. The FFQ captured the frequency of consuming various foods over the past year, with interviewees noting servings, grams, and consumption frequency (daily, weekly, or monthly). The consumption of each specific food and beverage was comprehensively recorded through the two 24 h recalls. Visual aids and instructions were provided to aid participants in accurately recalling food intake and estimating portion sizes.

#### 2.3.3. Phase 3: Anthropometric Measurements

During this stage, anthropometric measurements (weight and height) of the participants were taken at a designated center or facility within their respective governorates. Standardized protocols and calibrated equipment, including a digital scale for weight and a stadiometer for height, were employed to ensure precise measurements. To enhance accuracy, each participant’s height and weight were measured thrice, and the Body Mass Index (BMI) was calculated by averaging the three recorded readings.

### 2.4. Data Management and Data Analysis

Excel 2016 was used to code and organize the data. The CI was calculated by dividing the total number of people living in the home (apart from infants) by the total number of rooms (apart from bathrooms and kitchens) [16]. The food items were divided into food groups according to the classifications of the following three distinct diets: the ‘Mediterranean Diet’ [18], the ‘EAT-Lancet Diet’ [19,20], and the ‘USDA Diet’ [21]. The food group intake (g/day) was then calculated and compared to the recommendations of each of these three diets. To calculate the food group consumption (g/day), the following method was used: The daily consumption of each food item was determined using the FFQ data. To calculate the daily frequency (serving/day), a serving that was reported as being consumed on a weekly or monthly basis was divided by 7 or 30, respectively. Then, the quantity of each food item consumed (g/day) was calculated by multiplying the daily frequencies that resulted by the corresponding serving sizes of the individual food items (g/serving). After that, the individual foods were divided into categories according to the three diets. The total intake (g/day) for a food group was then determined by adding the quantities consumed of each food item belonging to the corresponding group.

After calculating the amounts of food consumed in grams per day, extraction of energy, macro- and micro-nutrients and the fiber content of the food consumed was undertaken using ‘Nutritionist Pro’ (version 5.1.0, 2014, First Data Bank, Nutritionist Pro, Axxya Systems, San Bruno, CA, USA), which is a software that permits the nutritional analysis of individual foods, menu items, and recipe ingredients [22]. The extracted nutritional value of food consumed was then compared to the age-specific Dietary Reference Intakes (DRIs), which were created by the ‘Food and Nutrition Board, Institute of Medicine, National Academies’ (NIH) and include the ‘Acceptable Macronutrient Distribution Range’ (AMDR), the ‘Adequate Intake’ (AI) and the ‘Recommended Dietary Allowance’ (RDAs) [23], ‘Dietary Cholesterol and Cardiovascular Risk: A Science Advisory From the American Heart Association’ [24], ‘Development of a Lebanese food exchange system based on frequently consumed Eastern Mediterranean traditional dishes and Arabic sweets’ [25], and ‘Nutritional value of the Middle Eastern diet: analysis of total sugar, salt, and iron in Lebanese traditional dishes’ [26]. Energy requirements were extracted from the ‘Dietary Guidelines for Americans 2020–2025’ [21].

### 2.5. Statistical Analysis

The sample was classified based on genders into 4 age categories in accordance with the ‘Food and Nutrition Board, Institute of Medicine, National Academies’ (NIH) [23] as follows: 18 years; 19–30 years; 31–50 years; and 51–64 years.

The Statistical Package for the Social Sciences (SPSS; Version 25.0, IBM Corp: Armonk, NY, USA) was used to analyze the data at a 95% confidence interval. Frequencies (N) and percentages (%) were calculated for categorical variables, while means and standard deviations (SD) were calculated for the continuous variables.

### 2.6. Ethical Considerations

The protocol for this study underwent review and approval by the Ethical Committee at Al Zahraa University Medical Center (#57/2022) and was carried out in compliance with the ethical principles set forth in the Declaration of Helsinki. Prior to participation, participants provided informed consent and were informed that their involvement was voluntary, with the option to withdraw at any point.

## 3. Results

### 3.1. Population Characteristics

The demographic and socioeconomic characteristics of the study population are shown in Table 1, and the study population’s health characteristics are shown in Table 2. Most participants were females (58.8%), and the mean age ± SD (years) was 34.1 ± 12.7. Most of the participants were residing in Mount Lebanon (40.32%), and most households were found to be crowded (62.84%), which reflects a lower socioeconomic status in these households compared to the non-crowded ones. Almost one-third of the participants (33.8%) had a normal BMI, while the majority (61.9%) were found to be overweight or obese. In addition, 25% of the participants reported having one or more chronic diseases, with anemia (32.4%) being the most prevalent disease, followed by hypertension (30.6%). Regarding the type of disease, more women were shown to be affected by the majority of diseases, except for kidney disease (similar between genders) and liver disease (more men are affected).

Significant differences between genders existed when it comes to residency (*p*-value = 0.006), crowding index (*p*-value = 0.028), occupation (*p*-value = 0.000), household income (*p*-value = 0.000), salary change (*p*-value = 0.000), the presence of chronic diseases (*p*-value = 0.005) and the disease type (*p*-value = 0.004).

### 3.2. Food Groups Consumption and Comparison to Different Diets’ Recommendations

#### 3.2.1. Food Groups Consumption

Mean intake of the different food groups by age and by gender are shown in Table 3 and Table 4, respectively. The description of food items included in each food group is shown in Table S1. In the overall study population, intake of bread/cereals/grains was the highest (317.18 g/d), followed by fruits (254.33 g/d) then vegetables (206.49 g/d). Among age categories, the mean intake of bread/cereals/grains was the highest compared to other food groups in all age categories except for participants in the 51–64 age group, in which the mean intake of fruits was the highest compared to other food groups (330 g/d). Significant differences in consumption existed among age categories when it comes to the consumption of processed meat (*p*-value = 0.000), poultry (*p*-value = 0.003), fresh fruit juices (*p*-value = 0.001), sweets (*p*-value = 0.000), and added fats/oils (*p*-value = 0.002). Regarding consumption based on gender, our results showed that, on average, male participants had a higher consumption compared to women from all the food groups except for vegetables, with significant differences in consumption existing when it comes to consuming nuts/seeds (*p*-value = 0.04), dairy products (*p*-value = 0.011), red meat (*p*-value = 0.000), processed meat (*p*-value = 0.000), poultry (*p*-value = 0.000), fish (*p*-value = 0.000), eggs (*p*-value = 0.000), drinking water (*p*-value = 0.000), non-alcoholic beverages (*p*-value = 0.000), and alcoholic beverages (*p*-value = 0.01).

#### 3.2.2. Comparison of Food Groups Consumed to the Mediterranean, the EAT-Lancet, and the USDA Diet Recommendations

**Figure 2:**
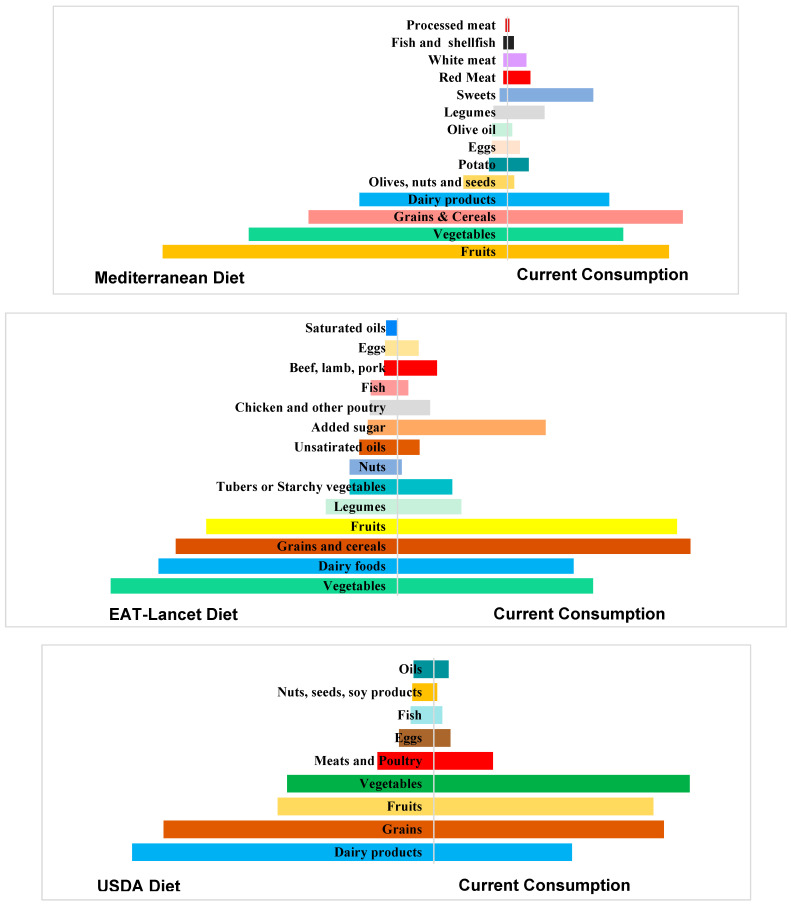
Comparison of the current food consumption to three different diets.

Pyramids showing comparisons of the current consumption to the Mediterranean, EAT-Lancet, and USDA diet recommendations are shown in Figure 2. In general, the dietary pattern of Lebanese adults showed low adherence to the recommendations of the three healthy diets.

For instance, the current consumption showed low adherence to the Mediterranean recommendations, especially the consumption of ‘sweets’, ‘red meat’, ‘white meat’, and ‘legumes’, for which consumption exceeded the recommended daily intake by 1106.4%, 516.8%, 427.5%, and 267.4%, respectively. Our results also showed that the consumption of ‘dairy products’, ‘fruits’, ‘vegetables’, ‘olive oil’, ‘olives/nuts/seeds’, and ‘grains/cereals’ was lower than the amounts recommended by the Mediterranean diet. As for the EAT-Lancet recommendations, the consumption of ‘added sugar’, ‘beef, lamb, pork’, ‘grains’, ‘chicken and other poultry’, and ‘fruits’ exceeded the recommended daily intake by 499.9%, 295%, 132%, 118%, and 146.16%, respectively. In addition, most of the consumed grains (92% of the amount consumed) are refined grains, which is far from this diet’s recommendation, as it recommends consuming 232 g/d of whole grains (compared to only 24 g/d in our study) and very low amounts (or nothing) of refined grains, in contrast to 274 g/d in our study. Our results also showed that the consumption of ‘vegetables’, ‘dairy products’, ‘fish’, ‘nuts’, and ‘unsaturated oils’ was lower than the amounts recommended by the EAT-Lancet diet. Regarding the USDA diet recommendations, the current consumption of ‘vegetables’, ‘fruits’ and ‘meat and poultry’ exceeded the recommendations by 174.37%, 146.17% and 104.57%, respectively. Additionally, a lower consumption of ‘grains’, ‘dairy products’, ‘fish’, ‘oils’, and ‘nuts, seeds, soy products’ was observed compared to the recommendations.

### 3.3. Energy Content of Food Consumed

The energy content of the food consumed by the study participants is shown in Table 5. The mean estimated energy requirement (EER) for a participant in our study was 2237.47 kcal/day, and a participant consumed on average 2237.49 kcal/day, which represents 100% of the mean EER. Our findings showed that, on average, participants of both genders and in all age categories exceeded their EER, except females in the ‘31–50 years’ age group, of which almost all consumed their EER (97%). Males and females in the ‘18 years’ age group had the highest consumption among male and female participants. Significant differences in the energy content of food consumed existed between genders (*p*-value = 0.000) and among age categories (*p*-value = 0.019).

### 3.4. Macronutrient Content of Food Consumed

The macronutrient content of food consumed by the study population is shown in Table 6. Our findings showed that the consumption of carbohydrates and fat exceeded the AMDR in participants from all age categories and genders, except the fat for the females in the 31–50 years group (97.4%). As for proteins, the AMDR was not reached in any of the age groups and genders, ranging from 65.3% for the female participants aged 31–50 years to 93.6% for the male participants aged 51–64 years. The consumption of monounsaturated fatty acids (MUFAs) did not exceed 60% of the RDA for all age categories and genders, except for males in the 51–64 years group (75.29%). In addition, participants from all age categories and genders exceeded the recommended daily limit of saturated fats, except for females in the 31–50 years group, who almost reached the recommended level (99.6%). Plus, the saturated fat content of food consumed exceeded 10% of the energy intake for males in the 19–30 years group and almost exceeded this limit for the other groups (>8% for all age groups and genders).

### 3.5. Micronutrient Content of Food Consumed

The micronutrient content of food consumed by the study population is shown in Table 7. Our results showed that participants in all age groups and genders had a low consumption of fat soluble vitamins (A, D, E), especially vitamin D, for which content in the consumed food was not 20% of the RDA. As for vitamin K, the RDA was exceeded for participants in all age groups and genders. Concerning water soluble vitamins, participants in all age groups and genders had a high consumption for all the vitamins except for biotin and B12. In general, the consumption of biotin did not exceed 85% of the RDA in all age groups and genders, and B12 consumption did not exceed 75% of the RDA in females belonging to the 51–64 years age group. In addition, our results revealed that females of reproductive age (belonging to the age groups 18, 19–30, 31–50) did not reach their daily requirements of iron, while females in all age categories had a low consumption of calcium, not exceeding 80% of the RDA in all the age categories. Moreover, our findings showed that the food consumed by Lebanese adults was high in sodium, with levels exceeding 150% of the RDA for all age groups and genders.

## 4. Discussion

This is the most updated study undertaken in Lebanon to assess the dietary consumption patterns of adults and report the energy, macro- and micro-nutrients of this consumption. Our results showed a low adherence to the following three different healthy diets: the Mediterranean, the EAT-Lancet, and the USDA diet. In addition, participants, especially women, failed to meet the RDA for many essential vitamins and minerals, notably vitamin D, calcium, and iron. Also, it was shown that Lebanese adults follow dietary patterns that are high in sodium, added sugars, and saturated fats, and low in potassium and MUFAs. Plus, the consumption of refined grains, red meat, and poultry exceeded the recommended amounts when compared to each of the three diets. A low consumption of seafood and nuts was observed which did not reach the recommendations when compared to each of the three diets. Our findings aligned with the results of a previous national study, which showed that Lebanese adults have low adherence to the Mediterranean diet [27]. Similarly in Italy [28], a study showed that Italian adult participants had a medium adherence to the Mediterranean diet, highlighting the need for public health policies to improve dietary habits. As for the EAT-Lancet diet, our findings align with a study conducted in Brazil [29] involving adults aged 20 years and above that showed a low adherence to the recommendations of this diet. The low adherence to the healthy and sustainable diets in our study population might be due to the shift in dietary patterns, as shown in a national study published in 2019 regarding the consumption patterns between the years 1997 and 2008/2009, which showed that the adult Lebanese population is shifting towards the Westernized dietary patterns and departing from the traditional Lebanese dietary pattern [30]. This low adherence to the healthy diets, particularly the Mediterranean diet, highlights the importance of shifting towards a healthier dietary pattern in this population, which can be achieved in many ways. For instance, Lebanese adults’ consumption of fruits, vegetables, nuts and seeds, and whole grains is lower than the recommended levels. In contrast, the consumption of red and processed meat, and sweets and added sugars exceeds the limits by far. Thus, reducing the consumption of sweets and red and processed meats and shifting to consuming more nuts, seeds, fruits, and vegetables can improve this population’s adherence to the Mediterranean and other healthy diets. In addition, Lebanese adults can replace red, processed and white meats with seafood, which will increase the consumption of the latter, as it was shown that consumption of fish and seafood, which contain healthy fats, is below the recommendations. These few changes can eventually improve the nutritional status of Lebanese adults, as fruits, vegetables and nuts/seeds/fish are rich in essential vitamins, minerals and healthy fats that are cardio-protective.

Our results showed that the food consumed by Lebanese adults is high in energy, with participants in all age groups and genders (except females in the 31–50 years group) exceeding their EER. This is reflected by most participants (61.9%) being overweight or obese. The following findings reveal that Lebanese adults are at a high risk of developing NR-NCDs, especially as the high-energy diet of this population is also very high in sodium and saturated fats and low in healthy fats (due to the low consumption of fish, nuts, and seeds). For instance, a systematic analysis conducted in 2017 showed that diets high in sodium are the leading cause of global deaths attributable to diet, while diets low in nuts and seeds were the fourth cause of global death attributable to diet, with the most deaths occurring from cardiovascular disease, followed by diabetes [31]. This is compounded by a low vitamin D intake for participants in all ages and genders and low iron and calcium intakes in female participants, which puts female Lebanese adults at a high risk of anemia and osteoporosis. For instance, women aged 50 years and above have a four times higher prevalence of osteoporosis and a two times higher prevalence of osteopenia in comparison to men [32], and low calcium intake is considered a modifiable risk factor for osteoporosis [33]. In addition, the amount of females of reproductive age (18–49 years) requiring higher amounts of iron and not meeting their requirements from food intake, as revealed in our study, is alarming, especially as 65.6% of the female participants in our study failed to meet their requirements. Concerning vitamin D deficiency, similar findings were found in Libya [34], Egypt [35], Iran [36], Qatar [37], and Saudi Arabia [38], where a high deficiency of vitamin D was prevalent within the populations. As for vitamins E, A, and K, our findings revealed that participants had a low intake of vitamins A and E and a high intake of vitamin K, which is consistent with a study performed in Jordan that showed that vitamin A and E daily dietary intakes were below the RDAs [39]. Furthermore, in Greece, a low nutrient intake of vitamin E was found in all age groups [40], while in Kuwait, around 80% of the population was shown to consume less than the RDAs of vitamins A and E [41]. Some studies showed different results when it comes to the consumption of vitamins A and E. A study conducted in Egypt revealed that the intakes of both vitamins A and E were within or above recommendations [35]. Similarly, in Pakistan, vitamin A intake seemed quite high at the national level [42]. The low consumption of vitamin A and E in our study might be due to the reduction in intake of animal products, fruit/vegetables for vitamin A and vegetable oils/nuts for vitamin E. As for vitamin B12, despite the mean intake being 2.9 μg/day, which is higher than the RDA (2.4 μg/day), and despite the mean intake for most age categories exceeding this RDA, more than half of the participants (52.5%) failed to meet this RDA, indicating a low intake in the adult population. Notably, in our study, younger adults (<30 years) were found to significantly consume more sweets and non-alcoholic beverages than older adults. This can be explained by younger generations being more willing to try new and trendy meals compared to older adults, who stick to old traditions [43].

When it comes to consumption patterns between the two genders, disparities may indeed exist. This could be attributed to various factors, such as cultural norms, access to resources, household dynamics, and societal roles. For instance, based on the ‘Food and Agriculture Organization of the United Nations’ (FAO), denying women’s rights is one of the leading causes of food and nutrition insecurity, making women more vulnerable to chronic nutrition and food insecurity [44]. Our findings align with studies that tackled gender disparities in food consumption. A study conducted in Bangladesh [45] showed that men had higher food intakes compared to females, as well as higher portion sizes. In addition, in Lebanon, a study showed that males had significantly higher energy intakes than females [46].

Compared to the findings of the nationally representative survey [30] that addressed the adult dietary consumption in the years 2008/2009 (see Table 8), our results showed a shift in the consumption of most of the food groups. For instance, an increased consumption of bread/cereals/grains (228 g/d vs. 317 g/d), legumes (40.87 g/d vs. 66.85 g/d), vegetables (190.93 g/d vs. 206.49 g/d), dairy products (88.21 g/d vs. 184.5 g/d), fruits (111.59 g/d vs. 254.33 g/d), fresh juices (8.53 mL/d vs. 38 mL/d), eggs (7.3 g/d vs. 22.23 g/d), chips and salty crackers (2.79 g/d vs. 8.35 g/d), hot beverages (217.75 mL vs. 546.83 mL/d), sweets (32.74 g/d vs. 65.36 g/d), and added sugars (7.3 g/d vs. 18.97 g/d) was observed. The consumption of red meat, processed meat, poultry, and fish underwent a slight change, while a significant decrease occurred in the consumption of nuts/seeds (9.44 g/d vs. 5 g/d), sugar sweetened beverages (165.76 mL/d vs. 75.86 mL/d), and alcoholic beverages (12.7 mL/d vs. 0.6 mL/d). This dietary shift in consumption occurred in both genders, with the consumption of a food group either increasing or decreasing simultaneously in both genders, except for vegetables consumption. In short, the increased consumption of fruits and vegetables and the decreased consumption of alcoholic beverages between the years 2009 and 2022 are considered positive shifts. However, this positive shift is faced by a negative shift characterized by decreased consumption of healthy fats, especially nuts and seeds, and a significant increase in the consumption of sweets, added sugars, sugar sweetened beverages, chips and salty crackers, and bread/cereals/grains, which include many refined items such as pasta, rice, and breakfast cereals. In addition, although the consumption of meat, poultry, and fish remained stable, the consumption of red meat remained higher than the limit set by the healthy diets, while the consumption of fish remained particularly low. This high consumption of red meat is alarming, since red meat is classified as a Group 2A carcinogen, meaning that it is “probably carcinogenic to humans”, with a 17% increase in the risk of developing cancer with every 100 g consumed per day [47]. Plus, excess red meat consumption is associated with higher risk of developing cardiovascular disease, type 2 diabetes and other NR-NCDs [47]. Moreover, the high consumption of sweets, especially in the younger adults’ generation, accompanied by an increased consumption of refined carbohydrates predispose Lebanese adults to increased adiposity levels and eventually overweight and obesity [48]. For instance, sweets and refined carbohydrates are energy-dense and low in proteins, fiber and essential macro- and micro-nutrients, and their high consumption is associated with increased risk of developing cardiometabolic diseases and dental caries, among other issues [48,49]. In short, our findings showed that Lebanese adults, especially women, are at high risk of developing NR-NCDs and hidden hunger.

## 5. Strengths and Limitations

This study has the major strength of being the most updated regarding assessing the dietary patterns of Lebanese adults and the corresponding nutritional value of these patterns following the economic crisis, providing valuable insights into the significance of this crucial matter. In addition, the study was performed on a nationally representative sample covering all the Lebanese governorates, which allows for the generalization of the results to the Lebanese adult population. However, this study has some limitations. The data collected in the study relied on self-reported data. This type of data is subject to inaccuracies due to recall bias, which might lead to inaccuracies in estimating portion sizes and under- and/or over-reporting of food consumption.

## 6. Conclusions

Our findings highlight unhealthy food consumption patterns in the Lebanese adult population characterized by high sodium, added sugars, and saturated fat intake, as well as low intakes of healthy fats, essential vitamins, and minerals, with the consumption of sweets and added sugars doubling compared to the year 2009. In a country where 91% of all deaths are attributed to NCDs [50], and where the prevalence of NR-NCDs is rising, these results are alarming, as the current dietary pattern has put the Lebanese adult population at high risk of developing NR-NCDs, NR-NCD complications, and hidden hunger. The findings of our study thus call for public health policies and interventions that allow for the adoption of healthy food choices and the shift towards healthier diets, such as the Mediterranean, the EAT-Lancet, or the USDA diet, which are proven to be healthy diets. This might require changes across the food system to focus on promoting healthier diets and ensuring their affordability, availability, accessibility, and acceptability for all [51].

## Figures and Tables

**Table 1:** Demographic and socioeconomic characteristics of the study population, overall and by gender.

KERRYPNX		Overall (*n* = 444)	Male (*n* = 183)	Female (*n* = 261)	*p*-Value
		*n* (%)	*n* (%)	*n* (%)	
Age Category	18 Years	20 (4.5%)	5 (25%)	15 (75%)	0.167
	19–30 Years	187 (42.1%)	78 (41.7%)	109 (58.3%)	
	31–50 Years	174 (39.2%)	79 (45.4%)	95 (54.6%)	
	51–64 Years	63 (14.2%)	21 (33.3%)	42 (66.7%)	
Residency (Governorate)	Akkar	31 (6.98%)	11 (35.5%)	20 (64.5%)	0.006 *
	Mount Lebanon	179 (40.32%)	73 (40.8%)	106 (59.2%)	
	Beqaa	27 (6.08%)	5 (18.5%)	22 (81.5%)	
	North Lebanon	60 (13.52%)	33 (55%)	27 (45%)	
	Baalbek-Hermel	20 (4.5%)	10 (50%)	10 (50%)	
	South Lebanon	55 (12.4%)	30 (54.5%)	25 (45.5%)	
	Beirut	32 (7.2%)	10 (31.2%)	22 (68.8%)	
	Nabatiyeh	40 (9%)	11 (27.5%)	29 (72.5%)	
Marital Status	Single	202 (45.5%)	81 (40.1%)	121 (59.9%)	0.139
	Married	223 (50.23%)	97 (43.5%)	126 (56.5%)	
	Widowed	7 (1.57%)	0 (0.0%)	7 (100%)	
	Divorced	12 (2.7%)	5 (41.7%)	7 (58.3%)	
Crowding index	No crowding	165 (37.16%)	79 (47.9%)	86 (52.1%)	0.028 *
	Crowding	279 (62.84%)	104 (37.3%)	175 (62.7%)	
Number of children	0	217 (48.87%)	87 (40.1%)	130 (59.9%)	0.124
	1–3	159 (35.81%)	74 (46.5%)	85 (53.5%)	
	>3	68 (15.32%)	22 (32.4%)	46 (67.6%)	
Education level	Illiterate	3 (0.68%)	1 (33.3%)	2 (66.7%)	0.961
	School	175 (39.41%)	72 (41.1%)	103 (58.9%)	
	University	266 (59.91%)	110 (41.4%)	156 (58.6%)	
Current Occupation	Unemployed	218 (49.1%)	40 (18.3%)	178 (81.7%)	0.000 *
	Employed	226 (50.9%)	143 (63.3%)	83 (36.7%)	
Monthly salary changes after economic crisis	No Impact	129 (29%)	63 (48.8%)	66 (51.2%)	0.000 *
	Decline in Salary	123 (27.7%)	51 (41.5%)	72 (58.5%)	
	Increase in Salary	70 (15.8%)	42 (60%)	28 (40%)	
	Already have no Salary	122 (27.5%)	27 (22.1%)	95 (77.9%)	
Household Monthly Income	None	39 (8.78%)	17 (43.6%)	22 (56.4%)	0.000 *
	Less than 1.5 million LBP	58 (13.1%)	15 (25.9%)	43 (74.1%)	
	>=1.5 million LBP	211 (47.5%)	79 (37.4%)	132 (62.6%)	
	<=300 USD	92 (20.72%)	41 (44.6%)	51 (55.4%)	
	More than 300 USD	44 (9.9%)	31 (70.5%)	13 (29.5%)	

* *p*-value < 0.05 is significant.

**Table 2:** Health characteristics of the study population, overall and by gender.

Variable		Overall (*n* = 444)		Male (*n* = 183)		Female (*n* = 261)		
		Mean	SD	Mean	SD	Mean	SD	*p*-Value
Weight (kg)		73.8	17.1	81.8	16.6	68.3	15.1	<0.001 *
Height (cm)		165.3	9.4	173.5	7	159.5	5.9	<0.001 *
Body mass index (kg/m^2^)		27	5.8	27.2	5.3	26.9	6.1	0.626
		*n*	%	*n*	%	*n*	%	
BMI classification	Underweight	19	4.3	4	21.1	15	78.9	0.084
	Normal	150	33.8	57	38	93	62	
	Overweight and Obese	275	61.9	122	44.4	153	55.6	
Disease status	No disease	333	75	150	45	183	55	0.005 *
	Having disease	111	25	33	29.7	78	70.3	
Disease type	Cardiovascular disease	14	12.6	5	15.2	9	11.5	0.004 *
	Diabetes	3	2.7	1	3	2	2.6	
	Hypertension	34	30.6	16	48.5	18	23.1	
	Kidney disease	4	3.6	2	6.1	2	2.6	
	Liver disease	1	0.9	1	3	0	0	
	Osteoporosis	14	12.6	1	3	13	16.7	
	Asthma/Respiratory diseases	12	10.8	2	6.1	10	12.8	
	Anemia	36	32.4	5	15.2	31	39.7	
	Others ^a^	32	28.8	6	18.2	26	33.3	

* *p*-value < 0.05 is significant. ^a^ Includes other self-reported diseases: (1) Allergies (seasonal, food, dust, skin); (2) vertebral column problems; (3) sarcoidosis; (4) migraine; (5) thyroid disease; (6) gastrointestinal problems; (7) psychological conditions; (8) neurological conditions; (9) hypovitaminosis D; (10) hypocalcemia; (11) iron deficiency; (12) urinary tract infection; (13) hypercholesterolemia; (14) Raynaud’s syndrome; (15) varicose veins; (16) autoimmune diseases; (17) cancer; (18) thrombosis; (19) polycystic ovarian syndrome.

**Table 3:** Food groups consumption by Lebanese adults, by age category.

	Dietary Intake (g/day)				
Food Groups	Overall (*n* = 444)				
	18 Years (*n* = 20)	19–30 Years (*n* = 187)	31–50 Years (*n* = 174)	51–64 Years (*n* = 63)	*p*-Value
Bread, Cereals and Grains	312 ± 89.78	329.88 ± 146.19	312.94 ± 132.31	292.61 ± 115.57	0.268
Legumes	73.03 ± 56.1	61.98 ± 59.97	67.95 ± 50.1	76.34 ± 50.01	0.297
Nuts and Seeds	2.56 ± 2.73	6.24 ± 16.52	3.84 ± 8.26	5.3 ± 9.37	0.246
Starchy Vegetables	43.5 ± 21.96	54.52 ± 49.02	48.21 ± 36.26	46.98 ± 48.94	0.391
Vegetables	177.86 ± 107.46	211.38 ± 118.85	194.73 ± 118.31	233.55 ± 112.69	0.087
Dairy Products	162.16 ± 128.4	201.19 ± 184.49	168.14 ± 150.94	187.19 ± 146.2	0.26
Meat and Meat Products, Poultry, Fish, Eggs					
Red Meat	39.35 ± 27.06	48.38 ± 52.85	36.73 ± 50.72	33.83 ± 26.19	0.069
Processed Meat	2.14 ± 2.46	4.71 ± 7.04	2.03 ± 3.17	1.66 ± 3.19	0.000 *
Poultry	30.96 ± 15.32	42.16 ± 41.42	30.62 ± 49.31	21.58 ± 20.28	0.003 *
Fish	7.97 ± 6.82	13.72 ± 26.05	10.08 ± 17.93	9.22 ± 13.45	0.244
Eggs	20.62 ± 19.47	26.58 ± 35.65	20.07 ± 34.12	15.84 ± 17.7	0.087
Fruits, Total					
Fruits	272.63 ± 229.84	231.99 ± 251.28	248.79 ± 293.12	330.09 ± 356.76	0.124
Fresh Juices (100% fruit juices) ^a^	52.39 ± 59.71	52.8 ± 95.58	29.84 ± 60.07	12.78 ± 24.97	0.001 *
Sweets and Added Sugars					
Sweets	178.53 ± 399.44	81.32 ± 93.52	44.77 ± 54.66	38.92 ± 52.19	0.000 *
Added Sugars, Jams, Honey, Molasses	23.75 ± 26.72	18.3 ± 22.36	18.9 ± 21.3	19.69 ± 22.66	0.761
Added Fats and Oils	14.15 ± 11.77	9.47 ± 9.44	11.89 ± 12.84	15.68 ± 13.13	0.002 *
Non-Alcoholic Beverages ^a^	104.6 ± 184.74	82.89 ± 127.27	60.99 ± 113.91	50.13 ± 87.95	0.093
Alcoholic Beverages ^a^	0 ± 0	0.41 ± 5.05	1.04 ± 7.79	0.19 ± 1.51	0.649

Values are Mean ± Standard deviation. * *p*-value < 0.05 is significant. ^a^ Beverages presented in mL/day.

**Table 4:** Food groups consumption by Lebanese adults, overall and by gender.

Food Groups	Dietary Intake (g/day)			
	Overall (*n* = 444)	Male (*n* = 183)	Female (*n* = 261)	*p*-Value
Bread, Cereals and Grains	317.18 ± 134.85	390.58 ± 139.96	265.71 ± 104.04	0.000
Legumes	66.85 ± 54.77	68.24 ± 62.25	65.89 ± 48.96	0.657
Nuts and Seeds	5 ± 12.46	6.45 ± 13.08	3.99 ± 11.93	0.04 *
Starchy Vegetables	50.48 ± 43.48	54.34 ± 42.38	47.78 ± 44.13	0.118
Vegetables	206.49 ± 117.79	197.76 ± 115.39	212.61 ± 119.29	0.191
Dairy Products	184.5 ± 164.72	208.33 ± 186.13	167.78 ± 145.94	0.011 *
Meat and Meat Products, Poultry, Fish, Eggs				
Red Meat	41.34 ± 48.39	54.45 ± 59.22	32.15 ± 36.47	0.000 *
Processed Meat	3.11 ± 5.32	4.35 ± 6.79	2.24 ± 3.76	0.000 *
Poultry	34.21 ± 42.34	46.55 ± 53.88	25.56 ± 28.98	0.000 *
Fish	11.39 ± 21.02	16.86 ± 28.22	7.56 ± 12.63	0.000 *
Eggs	22.23 ± 32.6	32.36 ± 44.33	15.13 ± 17.76	0.000 *
Fruits, Total				
Fruits	254.33 ± 284.95	275.91 ± 286.66	239.19 ± 283.32	0.182
Fresh Juices (100% fruit juices) ^a^	38 ± 75.5	40.58 ± 80.49	36.36 ± 71.94	0.563
Sweets and Added Sugars				
Sweets	65.36 ± 114.08	77.73 ± 159.17	56.69 ± 65.24	0.056
Added Sugars, Jams, Honey, Molasses	18.97 ± 22.15	20.47 ± 23.76	17.93 ± 20.94	0.235
Added Fats and Oils	11.51 ± 11.68	12.21 ± 13.75	11.02 ± 9.98	0.289
Herbs and Spices	47.87 ± 44.1	50.1 ± 46.08	46.31 ± 42.67	0.373
Hot Beverages ^a^	546.83 ± 427.36	543.56 ± 454.43	549.12 ± 408.19	0.893
Drinking Water ^a^	1440.48 ± 843.19	1819.37 ± 866.39	1236.91 ± 747.44	0.000 *
Non-alcoholic beverages ^a^	70.63 ± 120.94	97.04 ± 160.6	52.12 ± 77.66	0.000 *
Alcoholic beverages ^a^	0.6 ± 5.9	1.47 ± 9.14	0 ± 0	0.01 *

Values are Mean ± Standard deviation. * *p*-value < 0.05 is significant. ^a^ Beverages presented in mL/day.

**Table 5:** Energy content of food consumed by Lebanese adults and the corresponding percentage contribution to the estimated energy requirement per age and per gender.

Consumption					
Estimated Energy Intake	Age Group (*n* = 444)				*p*-Value *
	18 Years (*n* = 20)	19–30 Years (*n* = 187)	31–50 Years (*n* = 174)	51–64 Years (*n* = 63)	0.019
	Mean ± SD				
Energy Content of Food Consumed (Kcal)	M: 3032 ± 959 F: 2156 ± 760.78	M: 2830.6 ± 1397.8 F: 2153.9 ± 1437	M: 2490.5 ± 1184.69 F: 1751.5 ± 806.7	M: 2566.5 ± 662.8 F: 1746.1 ± 675.47	0.000
EER (kcal/d)	M: 2200 F: 1800	M: 2400 F: 2000	M: 2200 F: 1800	M: 2000 F: 1600	
% EER ^a^	M: 137.8% F: 119.78%	M: 117.92% F: 107.69%	M: 113.18% F: 97.3%	M: 128.3% F: 109.12%	

^a^ %EER = Energy Content/EER × 100. * *p*-value < 0.05 is significant. Abbreviations: d day, EER Estimated Energy Requirement, F female, kcal kilocalories, M male, n number of participants, SD Standard Deviation.

**Table 6:** Macronutrient content of food consumed by Lebanese adults and the percent contribution to daily value, per age and per gender.

**Age Group**	**18 Years (*n* = 20)**						**19–30 Years (*n* = 187)**					
**Gender**	**Male (*n* = 5)**			**Female (*n* = 15)**			**Male (*n* = 78)**			**Female (*n* = 109)**		
**Nutrient**	**DRI** **(RDA/** **AMDR)**	**Mean ± SD**	**% DV**	**DRI** **(RDA/** **AMDR)**	**Mean ± SD**	**% DV**	**DRI** **(RDA/** **AMDR)**	**Mean ± SD**	**% DV**	**DRI** **(RDA/** **AMDR)**	**Mean ± SD**	**% DV**
**Macronutrients**												
Total Fat (g/d)	**73**	95.18 ± 38.39	130.38%	**60**	75.78 ± 32.47	126.3%	**80**	96.53 ± 54.75	120.66%	**66.7**	75.75 ± 64.89	113.57%
Cholesterol (mg/d) ^a^	<300	344.83 ± 171.76	114.94%	<300	204.28 ± 92.56	68.1%	< 300	368.24 ± 274.83	122.75%	<300	237.72 ± 166.55	79.24%
Saturated fat (g/d) ^b^	22	32 ± 13.92	145.45%	18	22.96 ± 10.93	127.56%	24	31.74 ± 20.38	132.25%	20	22.81 ± 13.18	114.05%
Monounsaturated fat (g/d) ^b^	48.4	29.03 ± 14.54	59.98%	39.6	23.78 ± 10.33	60.05%	52.8	30.41 ± 17.32	57.59%	44	23.51 ± 17.62	53.43%
Polyunsaturated fat (g/d) ^b^	24.2	17.69 ± 6.63	73%	19.8	17.93 ± 9.06	90.56%	26.4	18.86 ± 11.27	71.44%	22	14.3 ± 9.16	65%%
Linoleic acid (g/d)	16	15.05 ± 7.04	94%	11	16.46 ± 8.69	149.63%	17	16.35 ± 10.35	96.18%	12	12.69 ± 8.52	105.75%
Alpha linolenic acid (g/d)	1.6	1.03 ± 0.27	64.37%	1.1	0.92 ± 0.35	83.63%	1.6	1.26 ± 0.77	78.75%	1.1	0.95 ± 0.54	86.36%
Trans Fat (g/d) ^b^	2.42	0.36 ± 0.23	14.87%	1.98	0.36 ± 0.45	18.18%	2.64	0.38 ± 0.35	14.39%	2.2	0.28 ± 0.28	12.73%
Carbohydrate (g/d)	**275**	455.8 ± 169.3	165.74%	**225**	310.5 ± 119.99	138%	**300**	390.1 ± 195	130%	**250**	300.3 ± 180.64	120.12%
Total sugar (g/d) ^c^	161.7	192.56 ± 154.85	119.08%	132.3	114 ± 76.54	86.17%	176.4	120.5 ± 90.41	68.3%	147	100.52 ± 73.3	68.38%
Protein (g/d)	**110**	97.82 ± 37.66	88.9%	**90**	68.1 ± 17.93	75.67%	**120**	106.55 ± 58.81	88.79%	**100**	76.32 ± 47.28	76.32%
Dietary fiber (g/d)	38	25.25 ± 11.09	66.45%	26	24.17 ± 9.67	92.96%	38	27.44 ± 15.47	72.21%	25	33.79 ± 105.35	135.16%
**As percentage of EI**												
Total Fat (%)	**25–30%**	27.89%	Within range	**25–30%**	31%	More than required	**20–35%**	30.42%	Within range	**20–35%**	31.15%	Within range
Saturated fat (%) ^b^	<10%	9.37%	Did not exceed	<10%	9.4%	Did not exceed	<10%	10%	Exceeded	<10%	9.38%	Did not exceed
Carbohydrate (%)	**45–65%**	59.37%	Within range	**45–65%**	56.6%	Within range	**45–65%**	54.65%	Within range	**45–65%**	54.9%	Within range
Total sugar (%)^c^	<30%	25%	Did not exceed	<30%	20.76%	Did not exceed	<30%	16.88%	Did not exceed	<30%	18.37%	Did not exceed
Protein (%)	**10–30%**	12.74%	Within range	**10–30%**	12.4%	Within range	**10–35%**	14.93%	Within range	**10–35%**	13.95%	Within range
**Age Group**	**31–50 years (*n* = 174)**						**51–64 Years (*n* = 63)**					
**Gender**	**Male (*n* = 79)**			**Female (*n* = 95)**			**Male (*n* = 21)**			**Female (*n* = 42)**		
**Nutrient**	**DRI** **(RDA/** **AMDR)**	**Mean ± SD**	**% DV**	**DRI** **(RDA/** **AMDR)**	**Mean ± SD**	**% DV**	**DRI** **(RDA/** **AMDR)**	**Mean ± SD**	**% DV**	**DRI** **(RDA/** **AMDR)**	**Mean ± SD**	**% DV**
**Macronutrients**												
Total Fat (g/d)	**73**	80.34 ± 46.37	110.05%	**60**	58.44 ± 36.74	97.4%	**66.7**	91.15 ± 42.85	136.66%	**53**	56.94 ± 25.82	107.44%
Cholesterol (mg/d) ^a^	<300	262.29 ± 222.5	60.76%	<300	179.9 ± 141.7	59.97%	<300	293.25 ± 116.63	97.75%	<300	139.82 ± 66.55	46.61%
Saturated fat (g/d) ^b^	22	23.67 ± 14.28	107.59%	18	17.93 ± 15.24	99.61%	20	26.58 ± 12.41	132.9%	16	16.14 ± 7.77	100.88%
Monounsaturated fat (g/d) ^b^	48.4	26.48 ± 18.85	54.71%	39.6	17.74 ± 11.84	44.8%	44	33.13 ± 19.22	75.29%	35.2	18.47 ± 9.56	52.47%
Polyunsaturated fat (g/d) ^b^	24.2	17.08 ± 11.61	70.58%	19.8	12.76 ± 7.83	64.44%	22	18.91 ± 10.16	85.95%	17.6	13.15 ± 7.48	74.71%
Linoleic acid (g/d)	17	15.22 ± 10.56	89.53%	12	11.47 ± 7.18	95.58%	14	16.32 ± 9.06	116.57%	11	11.69 ± 6.79	106.27%
Alpha linolenic acid (g/d)	1.6	1.1 ± 1.01	68.75%	1.1	0.81 ± 0.58	73.64%	1.6	1.32 ± 0.95	82.5%	1.1	0.75 ± 0.37	68.18%
Trans Fat (g/d) ^b^	2.42	0.32 ± 0.43	13.22%	1.98	0.19 ± 0.15	9.59%	2.2	0.24 ± 0.2	10.91%	1.76	0.18 ± 0.13	10.23%
Carbohydrate (g/d)	**275**	353.93 ± 170	128.7%	**225**	254.5 ± 107.9	113.11%	**250**	351.58 ± 71.5	140.63%	**200**	261.24 ± 110.53	130.62%
Total sugar (g/d) ^c^	161.7	98.27 ± 67.59	60.77%	132.3	81.26 ± 60.63	61.42%	147	99.4 ± 42.86	67.62%	117.6	86.97 ± 61.44	73.95%
Protein (g/d)	**110**	94.36 ± 59.89	85.78%	**90**	58.77 ± 27.56	65.3%	**100**	93.61 ± 31.44	93.6%	**80**	56.47 ± 19.51	70.59%
Dietary fiber (g/d)	38	29.54 ± 27.66	77.74%	25	21.31 ± 9.36	85.24%	30	29.22 ± 9.93	97.4%	21	25.24 ± 10.82	120.19%
**As percentage of EI**												
Total Fat (%)	**20–35%**	29%	Within range	**20–35%**	29.56%	Within range	**20–35%**	31.5%	Within range	**20–35%**	28.74%	Within range
Saturated fat (%) ^b^	<10%	8.47%	Did not exceed	<10%	9.07%	Did not exceed	<10%	9.2%	Did not exceed	<10%	8.14%	Did not exceed
Carbohydrate (%)	**45–65%**	56%	Within range	**45–65%**	57.22%	Within range	**45–65%**	54.1%	Within range	**45–65%**	58.6%	Within range
Total sugar (%) ^c^	<30%	15.62%	Did not exceed	<30%	18.27%	Did not exceed	<30%	15.28%	Did not exceed	<30%	19.51%	Did not exceed
Protein (%)	**10–35%**	15%	Within range	**10–35%**	13.21%	Within range	**10–35%**	14.4%	Within range	**10–35%**	12.66%	Within range

Abbreviations: AMDR Acceptable Macronutrient Distribution Range, d day, DRI Dietary Reference Intake, DV Daily Value, g grams, n number of participants, RDA Recommended Dietary Allowance, SD Standard Deviation. Values in bold are AMDR (50% carbohydrates, 20% proteins, 30% fat) based on the estimated energy requirement of each age group. ^a^ [24,25,26], ^b,c^ Values were extracted based on a 2000 kcal-diet then adjusted to each age group’s estimated energy requirements.

**Table 7:** Micronutrient content of food consumed by Lebanese adults and the percent contribution to daily value per age and gender.

**Age Group**	**18 Years**						**19–30 Years**					
**Gender**	**Male**			**Female**			**Male**			**Female**		
**Nutrient**	**DRI** **(RDA/AI)**	**Mean ± SD**	**%DV**	**DRI** **(RDA/AI)**	**Mean±SD**	**%DV**	**DRI** **(RDA/AI)**	**Mean±SD**	**%DV**	**DRI** **(RDA/AI)**	**Mean ± SD**	**%DV**
**Antioxidants**												
Vitamin C (mg/d)	75	129.8 ± 75.82	173%	65	116.31 ± 87.1	178.94%	90	130.6 ± 116.18	145.11%	75	122.1 ± 155.26	162.67%
**B Vitamins**												
Thiamin (mg/d)	1.2	1.38 ± 0.43	115%	1	1.08 ± 0.3	108%	1.2	1.57 ± 0.79	130.83%	1.1	1.11 ± 0.51	100.91%
Riboflavin (mg/d)	1.3	1.64 ± 0.57	126.15%	1	1.19 ± 0.4	119%	1.3	1.85 ± 1.14	142.31%	1.1	1.35 ± 0.85	122.73%
Niacin (mg/d)	16	22.44 ± 5.78	140.25%	14	16.8 ± 5.17	120%	16	29.1 ± 20.93	181.87%	14	17.95 ± 9.27	128.2%
Vitamin B-6 (mg/d)	1.3	1.79 ± 0.67	137.69%	1.2	1.78 ± 1.03	148.33%	1.3	2.84 ± 3.41	218.46%	1.3	1.62 ± 0.92	124.61%
Folate (μg dietary folate equivalent/d)	400	432.81 ± 191.86	108.2%	400	355.6 ± 107.4	88.91%	400	448.53 ± 247.1	121.13%	400	364.85 ± 177.65	91.21%
Vitamin B-12 (μg/d)	2.4	3 ± 1.3	125%	2.4	2.39 ± 1.09	99.58%	2.4	4.41 ± 3.96	183.75%	2.4	2.75 ± 1.99	114.58%
Biotin (µg/d) *	25	20.97 ± 9.15	83.88%	25	15.28 ± 9.14	61.12%	30	23.41 ± 17.5	78%	30	18.53 ± 20.69	61.77%
Pantothenic Acid (mg/d) *	5	5.1 ± 1.55	102%	5	4.53 ± 1.55	90.6%	5	6.43 ± 4.42	128.6%	5	4.6 ± 2.48	92%
**Bone-Related Nutrients**												
Calcium (mg/d) *	1300	1023.4 ± 334.54	78.72%	1300	701.25 ± 233	53.94%	1000	1086.1± 567.55	108.6%	1000	761.17 ± 321.66	76.11%
Phosphorus (mg/d)	1250	1325 ± 496.28	106%	1250	903.8 ± 254.2	72.3%	700	1366.8 ± 811.48	195.26%	700	990.24 ± 510.5	141.46%
Magnesium (mg/d)	410	394 ± 58.6	96.1%	360	291.6 ± 82.83	81%	400	421.49 ± 225.55	105.37%	310	319.21 ± 147.85	102.97%
Vitamin D (IU/d) *	600	84 ± 53.6	14%	600	46.8 ± 34.8	7.8%	600	100 ± 109.6	16.67%	600	67.6 ± 58.4	9.73%
**Other Micronutrients**												
Vitamin A (μg retinol activity equivalent/d)	900	448.9 ± 219.86	49.88%	700	435 ± 260.46	62.14%	900	595.88 ± 548.11	66.21%	700	511.1 ± 1295.87	73%
Vitamin E of alpha-Tocopherol Equivalents (mg/d)	15	8.32 ± 4.77	55.47%	15	9.36 ± 5.3	62.4%	15	8.58 ± 5.45	57.2%	15	7.37 ± 5.57	49.13%
Vitamin K (μg/d) *	75	123 ± 54.23	164%	75	100.9 ± 50.49	134.53%	120	124.38 ± 84.03	103.65%	90	103.76 ± 71.03	115.29%
Iron (mg/d)	11	18.81 ± 6.92	171%	15	13.45 ± 4.21	89.67%	8	18.21 ± 8.93	227.62%	18	14.84 ± 18.99	82.44%
Zinc (mg/d)	11	9.97 ± 3.36	90.64%	9	8.4 ± 3.1	93.33%	11	11.87 ± 7.4	107.91%	8	8.81 ± 5.02	110.25%
Sodium (g/d) *	1.5	3.99 ± 1.19	266%	1.5	2.88 ± 0.95	192%	1.5	4.04 ± 1.92	269.33%	1.5	3.37 ± 4.93	224.67%
Potassium (g/d) *	3.4	2.86 ± 0.86	84.11%	3.4	2.5 ± 0.99	73.53%	3.4	3.1 ± 1.72	91.17%	3.4	2.63 ± 1.28	77.35%
Copper (mcg/d)	890	1730 ± 650	194.38%	890	1410 ± 340	158.43%	900	2000 ± 1300	222.22%	900	1700 ± 2480	188.89%
Manganese (mg/d) *	2.2	4.17 ± 1.92	189.54%	1.6	3.21 ± 0.9	200.6%	2.3	4.32 ± 2.68	187.83%	1.8	3.57 ± 2.15	198.33%
Selenium (µg/d)	55	61.49 ± 24.52	111.8%	55	42.9 ± 16.59	78%	55	79.64 ± 58.51	144.8%	55	53.48 ± 34.49	97.24%
Fluoride (mg/d) *	3	1.82 ± 1.34	60.67%	3	0.86 ± 0.67	28.67%	4	1.33 ± 1.457	33.25%	3	1.76 ± 2.15	58.67%
Chromium (mcg/d) *	35	30 ± 20	85.71%	24	20 ± 20	83.33%	35	30 ± 30	85.71%	25	20 ± 40	80%
Molybdenum (µg/d)	43	17.43 ± 8.68	40.53%	43	18.88 ± 13.75	43.91%	45	21.36 ± 18.44	47.47%	45	17.83 ± 15.35	39.62%
**Age Group**	**31–50 years**						**51–64 years**					
**Gender**	**Male**			**Female**			**Male**			**Female**		
**Nutrient**	**DRI** **(RDA/AI)**	**Mean ± SD**	**%DV**	**DRI** **(RDA/AI)**	**Mean ± SD**	**%DV**	**DRI** **(RDA/AI)**	**Mean ± SD**	**%DV**	**DRI** **(RDA/AI)**	**Mean ± SD**	**%DV**
**Antioxidants**												
Vitamin C (mg/d)	90	110.63 ± 98.1	192.22%	75	86.56 ± 79.39	115.41%	90	123.49 ± 63.46	137.21%	75	110.68 ± 110.75	147.57%
**B Vitamins**												
Thiamin (mg/d)	1.2	1.42 ± 0.68	118.33%	1.1	0.96 ± 0.4	87.27%	1.2	1.5 ± 0.4	125%	1.1	0.98 ± 0.38	89.1%
Riboflavin (mg/d)	1.3	1.68± 0.86	129.23%	1.1	1.2 ± 0.76	109.1%	1.3	1.81 ± 0.5	139%	1.1	1.17 ± 0.47	106.36%
Niacin (mg/d)	16	25.8 ± 15.59	161.25%	14	15.68 ± 9.41	112%	16	25.43 ± 10.39	158.94%	14	16.29 ± 7.3	116.35%
Vitamin B-6 (mg/d)	1.3	1.8 ± 1.38	138.46%	1.3	1.26 ± 0.85	96.92%	1.7	2.01 ± 1.27	118.23%	1.5	1.32 ± 0.72	88%
Folate (μg dietary folate equivalent/d)	400	471.12 ± 555.79	117.78%	400	330.59 ± 148.51	82.65%	400	429.98 ± 112.59	107.49%	400	359.66 ± 155.74	89.91%
Vitamin B-12 (μg/d)	2.4	3.21 ± 2.42	133.75%	2.4	2.12 ± 1.71	88.33%	2.4	3.66 ± 2.12	152.5%	2.4	1.78 ± 1.02	74.17%
Biotin (µg/d) *	30	19.85 ± 17.08	66.17%	30	13.34 ± 10.34	44.47%	30	23.64 ± 11.99	78.8%	30	13.67 ± 10.29	45.57%
Pantothenic Acid (mg/d) *	5	5.14 ± 2.69	102.8%	5	3.83 ± 2.25	76.6%	5	5.43 ± 1.52	108.6%	5	3.6 ± 1.53	72%
**Bone-Related Nutrients**												
Calcium (mg/d) *	1000	962.51 ± 420.83	96.25%	1000	701.82 ± 359.83	70.18%	1000	1110.3 ± 379.9	111%	1200	659 ± 234.1	54.92%
Phosphorus (mg/d)	700	1200.3 ± 760.1	171.47%	700	813.97 ± 406.04	116.28%	700	1234.3 ± 432.1	176.33%	700	771.55 ± 286.14	110.22%
Magnesium (mg/d)	420	450 ± 265.6	107.14%	320	318.34 ± 156.1	99.48%	420	477.81 ± 123.6	113.76%	320	341.52 ± 140	106.72%
Vitamin D (IU/d) *	600	71.2 ± 71.6	11.87%	600	50 ± 60	8.33%	600	80 ± 58	13.33%	600	39.2 ± 34.8	6.53%
**Other Micronutrients**												
Vitamin A (μg retinol activity equivalent/d)	900	484.83 ± 432.1	53.87%	700	357 ± 317.8	51%	900	613.12 ± 349.17	68.12%	700	320 ± 207.6	45.71%
Vitamin E of alpha-Tocopherol Equivalents (mg/d)	15	8.9± 6.1	59.33%	15	6.41 ± 4.46	42.73%	15	10.26 ± 5.42	68.4%	15	7.23 ± 4.66	48.2%
Vitamin K (μg/d) *	120	124.81 ± 106.97	104%	90	91.69 ± 62.7	101.88%	120	206.59 ± 206.23	172.16%	90	100.35 ± 53.89	111.5%
Iron (mg/d)	8	17.36 ± 11.76	217%	18	11.54 ± 4.74	64.11%	8	17.43 ± 4.96	217.87%	8	12.66 ± 5.22	158.25%
Zinc (mg/d)	11	10.82 ± 7.33	98.36%	8	7.31 ± 3.7	91.37%	11	11.05 ± 3.86	100.45%	8	7.05 ± 2.55	88.12%
Sodium (g/d) *	1.5	3.62 ± 1.69	241.33%	1.5	2.5 ± 0.99	166.67%	1.3	3.7 ± 0.91	284.61%	1.3	2.49 ± 0.81	191.5%
Potassium (g/d) *	3.4	3.03 ± 1.87	89.12%	3.4	2.26 ± 1.16	66.47%	3.4	3.25 ± 0.88	95.59%	3.4	2.46 ± 1.22	72.35%
Copper (mcg/d)	900	2010 ± 1400	223.33%	900	1370 ± 560	152.22%	900	1990 ± 580	221.11%	900	1380 ± 540	153.33%
Manganese (mg/d) *	2.3	4.66 ± 3.95	202.61%	1.8	3.17 ± 1.47	176.11%	2.3	4.86 ± 1.58	211.3%	1.8	3.52 ± 1.68	195.56%
Selenium (µg/d)	55	65.1 ± 54.57	118.36%	55	36.46 ± 24.1	66.3%	55	64.29 ± 34.2	116.89%	55	33.18 ± 16.23	60.33%
Fluoride (mg/d) *	4	2.04 ± 2.73	51%	3	4.02 ± 8.99	134%	4	3.05 ± 2.57	76.25%	3	2.97 ± 3.1	99%
Chromium (mcg/d) *	35	30 ± 30	85.71%	25	10 ± 10	40%	30	30 ± 20	100%	20	10 ± 10	50%
Molybdenum (µg/d)	45	19.62 ± 17.31	43.6%	45	16.88 ± 14.88	37.51%	45	29.48 ± 18.23	65.51%	45	17.72 ± 15.14	39.38%

Abbreviations: AI Adequate Intake, d day, DRI Dietary Reference Intake, DV Daily Value, g grams, n number of participants, RDA Recommended Dietary Allowance, SD Standard Deviation. * Values are AIs.

**Table 8:** Comparison of the current adult food-group consumption to the consumption of the year 2009.

Food Groups	Mean Dietary Intake (g/d)					
	Males (*n* = 1319)		Females (*n* = 1643)		Overall (*n* = 2962)	
	2009	2022	2009	2022	2009	2022
Bread	148.27	183.01	79.3	146.73	109.18	161.68
Cereals and cereal based products	145.88	207.56	96.49	118.98	119.08	155.5
Legumes	53.99	68.24	30.8	65.89	40.87	66.85
Nuts and Seeds	10.97	6.45	8.35	3.99	9.44	5
Starchy Vegetables	42.78	46.01	29.56	39.42	35.34	42.13
Vegetables	209.87	197.76	176.3	212.61	190.93	206.49
Dairy Products	95.94	208.33	82.16	167.78	88.21	184.5
Meat and Meat Products, Poultry, Fish, Eggs						
Red Meat	58.01	54.45	29.96	32.15	42.26	41.34
Processed Meat	5.99	4.35	3.27	2.24	4.49	3.11
Poultry	38.68	46.55	21.02	25.56	29.07	34.21
Fish	18.09	16.86	8.44	7.56	12.78	11.39
Eggs	10.75	32.36	4.76	15.13	7.3	22.23
Fruits, Total						
Fruits	115.96	275.91	108.29	239.19	111.59	254.33
Fresh Juices (100% fruit juices) *	9.48	40.58	7.7	36.36	8.53	38
Chips and Salty Crackers	2.98	8.33	2.73	8.36	2.79	8.35
Sweets and Added Sugars						
Sweets	33.54	77.73	32.16	56.69	32.74	65.36
Added Sugars, Jams, Honey, Molasses	8.56	20.47	5.99	17.93	7.08	18.97
Added Fats and Oils	21.12	12.21	10.39	11.02	15.1	11.51
Hot Beverages *	198.7	543.56	237.51	549.12	217.75	546.83
Sugar Sweetened Beverages *	171.44	96.18	156.85	62.49	165.76	75.86
Alcoholic Beverages *	23.84	1.47	4.02	0	12.7	0.6

* Values in mL/day.

## Data Availability

The original contributions presented in the study are included in the article, further inquiries can be directed to the corresponding author.

## Supplementary Materials

The following supporting information can be downloaded at: https://www.mdpi.com/article/10.3390/nu16111784/s1, Table S1: Food items included in each food group.

## Appendix A

Adults-LEBANON-FCS group: Zahraa Fadlallah, Razan Khadra (Faculty of Public Health, Lebanese University); Mohamad Chahine, Omasyarifa Binti Jamal Poh (Kursk State University, Russia).
