# Supplementary material for: Dietary Intake among Lebanese Adults: Findings from the Updated LEBANese natiONal Food Consumption Survey (LEBANON-FCS)
Source: Nutrients. 2024 Jun 6;16(11):1784. doi: 10.3390/nu16111784 (PMC11174657; doi:10.3390/nu16111784)
Supplement: Supplementary file 1 [file nutrients-16-01784-s001.zip › nutrients-3044936-supplementary.pdf]

Table S1. Food items included in each food group.

**Food Groups**	**Items**
Bread, Grains, Cereals and Cereal-Based Products	All kinds of breads, Cereals, rice, rice-based dishes, pasta, oat, bulgur, Lebanese kaake, quinoa, kechek
Legumes	All kinds of legumes, legume-based dishes, peas and beans
Starchy Vegetables	Potatoes (including potato-based dishes), corn, chips, pop corn
Vegetables	Raw vegetables (including all kinds of vegetables and salads), cooked vegetables, pickled vegetables, vegetable-based traditional dishes, tomato paste, vegetables soups
Nuts and Seeds	All kinds of nuts and seeds (including almonds, cashew nuts, pistachios, walnuts, pine nut, hazelnut, coconut, seeds)
Dairy Products	All kinds of milk<br>All kinds of cheese, yogurt and yogurt-based dishes, laban, kariche<br>Milk-based dishes, puddings, frozen and fruit yogurt
Meat, Processed Meat, Poultry, Fish, Eggs	
Red Meat	Meat and organ meats
Processed Meat	Processed Meat (hotdog, salami, mortadella)
Poultry	Poultry and poultry organs (chickens, birds)
Fish	All kinds of seafood (tuna, sardines, fish)
Eggs	Eggs
Fruits, Total	
Fruits	All fruits, fruit salads and dried fruits
Fresh Fruit Juices	Juices made from 100% fruits
Sweets and Added Sugars	
Sweets	Pastries, candies, biscuits, cakes, traditional sweets (Jello, custard, Arabic sweets), ice cream
Added Sugars	Added sugars, jams, honey, molasses
Hot Beverages	Coffee, tea, Nescafe with/out coffee mate, iced tea
Non-alcoholic Beverages	Sweetened juices, regular soft drinks
Alcoholic Beverages	Spirits and alcohols
Added Fats and Oils	Olive oil, olives, avocado, margarine, butter, all kinds of oils (palm, sunflower, canola, coconut, corn), animal-based fat
